# How does dispositional mindfulness foster prosocial behavior? A cross-cultural study of empathy’s mediating role and cultural moderation

**DOI:** 10.3389/fpsyg.2024.1451138

**Published:** 2024-11-20

**Authors:** Sisi Li, Nailiang Zhong, Qingke Guo

**Affiliations:** ^1^Department of Psychology, Guangxi Normal University, Guilin, China; ^2^Liru Senior High School, South China Normal University, Xinyi, China; ^3^School of Teacher Education, Hechi University, Hechi, China; ^4^School of Psychology, Shandong Normal University, Jinan, China

**Keywords:** dispositional mindfulness, empathy, prosocial behavior, cultural differences, mediating effect, moderating effect, cross-cultural research, perspective taking

## Abstract

**Background:**

Despite growing recognition of dispositional mindfulness (DM) in psychological research, its cross-cultural mechanisms in promoting prosocial behavior remain unclear, particularly regarding the mediating role of different empathy dimensions.

**Purpose:**

This study investigated how DM influences prosocial behavior across cultural contexts, examining both the mediating effects of different empathy dimensions and the moderating role of cultural background in Chinese and Indonesian samples.

**Methods:**

Participants included 683 university students (357 Chinese, 326 Indonesian) who completed the Mindful Attention Awareness Scale (MAAS), Interpersonal Reactivity Index (IRI), and Self-Report Altruism Scale Distinguished by the Recipient (SRAS-DR). Mediation and moderation analyses were conducted using PROCESS macro.

**Results:**

Chinese participants demonstrated higher DM levels than Indonesian participants. In the Chinese sample, both perspective taking (PT) and empathic concern (EC) mediated DM’s effects on prosocial behavior toward family (PBF), friends (PBFr), and strangers (PBS). However, in the Indonesian sample, PT and EC only mediated effects on PBFr. Cultural background significantly moderated DM’s indirect effect on PBS through PT, with stronger effects in the Chinese sample. Fantasy and personal distress showed no significant mediating effects in either cultural context.

**Conclusion:**

This study reveals that DM enhances prosocial behavior through selective influence on different empathy dimensions, with cultural background moderating specific pathways. These findings extend our understanding of mindfulness’s cross-cultural mechanisms and provide practical implications for culturally-adapted mindfulness interventions.

## 1 Introduction

Mindfulness, a psychological state rooted in Eastern traditions, has garnered substantial attention in recent years across psychology, neuroscience, and related fields. It is commonly defined as a state of conscious, non-judgmental attention to present-moment experiences (Kabat-Zinn, 2013a). Dispositional mindfulness refers to an individual’s natural tendency to maintain this attitude of awareness and acceptance in daily life (Brown and Ryan, 2003). Extensive research has demonstrated that dispositional mindfulness is associated with various positive psychological and behavioral outcomes, including reduced stress and anxiety (Khoury et al., 2013), enhanced subjective well-being (Keng et al., 2011), improved cognitive function (Chiesa et al., 2011), and increased prosocial behavior (Brown and Ryan, 2003; Berry et al., 2018). Prosocial behavior, defined as voluntary actions intended to benefit others, constitutes a fundamental basis for social harmony and human progress (Eisenberg, 1989). It encompasses behaviors such as helping, sharing, comforting, and cooperating, which are crucial for maintaining social cohesion and promoting positive interpersonal interactions. In an increasingly complex and interdependent global environment, understanding and promoting prosocial behavior has become particularly significant.

### 1.1 Mindfulness and prosocial behavior

In recent years, researchers have begun to examine the connection between mindfulness and prosocial behavior, discovering that mindfulness not only enhances individual psychological well-being but may also strengthen prosocial tendencies (Condon et al., 2013; Lim et al., 2015). Some scholars suggest that mindfulness can improve emotional regulation capacity, enabling individuals to experience more positive emotions and fewer negative ones, thereby promoting helping behavior (Raugh and Strauss, 2024; Remskar et al., 2024). Additionally, mindfulness training can lead to self-transcendence and the dissolution of self-centered focus (Hanley et al., 2020, 2023). The dissolution of boundaries between self and others facilitates prosocial behavior aimed at enhancing others’ well-being, as individuals begin to view others as extensions of themselves (Hölzel et al., 2011; Ma et al., 2024). However, although existing research has explored the relationship between dispositional mindfulness and prosocial behavior, this relationship’s manifestation across different cultural contexts remains understudied. Culture, as a shared system of beliefs, values, and behavioral patterns, profoundly influences individual cognition, emotion, and behavior (Markus and Kitayama, 1992). Cross-cultural psychological research indicates that cultural values, religious beliefs, and social norms may moderate the relationship between psychological characteristics and behavior (Matsumoto and Hwang, 2021). Therefore, investigating how the relationship between dispositional mindfulness and prosocial behavior manifests across different cultural contexts not only enhances our understanding of this relationship but also provides important insights for the application of mindfulness in cross-cultural settings.

### 1.2 The mediating role of empathy

Empathy, as a crucial psychological mechanism, is considered a significant mediating variable connecting dispositional mindfulness and prosocial behavior (Berry et al., 2020). Empathy encompasses both cognitive and affective dimensions, involving understanding others’ thoughts and experiencing others’ emotions, respectively (Davis, 1983). Research indicates that mindfulness can promote empathy by reducing self-centeredness and enhancing sensitivity to others’ needs (Condon et al., 2013). Mindfulness practice can help individuals better recognize their own emotional states, and this self-awareness may translate into a more acute understanding of others’ emotions (Borghi et al., 2023). Furthermore, mindfulness can facilitate more responsive reactions to others’ needs by reducing stress responses (Knudsen et al., 2023). However, the expression and function of empathy may be influenced by cultural context. For instance, in collectivist cultures, empathy may manifest more as concern for group harmony, while in individualistic cultures, it may be more oriented toward understanding individual needs (Chopik et al., 2016). Chinese Confucian tradition emphasizes the concept of “ren” (benevolence), and this form of care and compassion for others may influence how Chinese people express and experience empathy (Guo et al., 2021). In contrast, Indonesia’s Islamic tradition emphasizes the concept of ummah (Muslim community), which may influence the expression of empathy, manifesting it more as concern for the entire community (Osili and Ökten, 2015).

### 1.3 The present study

This study selects China and Indonesia for comparison, as these two countries exhibit both similarities and significant differences in their cultural backgrounds. In terms of similarities, both are developing Asian nations characterized by strong collectivist orientations and long-term perspectives (Hofstede et al., 2010). These collectivist tendencies may influence how individuals understand and practice mindfulness and prosocial behavior. For instance, in collectivist cultures, mindfulness may be viewed not only as awareness of personal internal experiences but also as attention to social relationships and group harmony (Pagis and Tal, 2022). Similarly, prosocial behavior in collectivist cultures may be perceived more as an obligation to maintain group welfare rather than merely a personal choice (Triandis, 2001; Aaldering et al., 2024).

However, China and Indonesia exhibit notable differences in their religious backgrounds and cultural traditions. Historically, China has been profoundly influenced by Confucianism, Buddhism, and Taoism, known as “the three teachings” (sanjiao), which became intertwined throughout historical development. Although modern Chinese society has become highly secularized, Buddhist thought continues to leave a deep imprint on the culture (Yang and Xu, 2023). Buddhism emphasizes awareness, compassion, and wisdom, concepts closely tied to mindfulness and prosocial behavior. For example, the Buddhist concept of “compassion” emphasizes sympathy and kindness toward all beings, which may promote prosocial behavior (Hamilton, 1950; Qin and Song, 2020; Makransky, 2021). Similarly, the Taoist concept of “clarity and tranquility” (qingjing) significantly influences Chinese psychological experience and behavior. This concept emphasizes achieving mind–body harmony through inner mental clarity and awareness, which closely aligns with mindfulness’s emphasis on present-moment awareness. The Confucian tradition emphasizes self-cultivation practices such as “self-restraint and returning to propriety” (keji fuli) and “vigilance in solitude” (shendu), forms of continuous self-reflection and behavioral awareness that share an intrinsic connection with the spirit of mindfulness.

In contrast, Indonesia, where approximately 87% of the population practices Islam, is the world’s largest Muslim nation, with Islam holding a dominant position in its sociocultural fabric (Islam in Indonesia, 2024). While Islam does not have an explicit concept of “mindfulness,” it contains similar practices such as dhikr (remembrance of Allah), muraqabah (contemplation), and Khushu (concentration). These practices similarly emphasize focus and self-awareness (Dwidiyanti et al., 2018). Additionally, Islam emphasizes charitable giving (zakat) and voluntary charity (sadaqah), teachings that may influence Muslims’ prosocial behavior (Lambarraa and Riener, 2015).

These cultural differences may influence the manifestation of dispositional mindfulness and its relationship with prosocial behavior in both countries. For example, while mindfulness originates from Buddhist tradition, modern mindfulness practices have been secularized, removing explicit religious elements (Kabat-Zinn, 2013b). However, some researchers argue that Buddhist ethics remain implicit in mindfulness practices (Purser and Milillo, 2014). In China, the Buddhist concepts of awareness, Taoist notions of clarity, and Confucian traditions of self-reflection form an interconnected cultural system. This diverse yet integrated spiritual tradition may make it easier for Chinese people to understand and accept mindfulness concepts, potentially leading to higher levels of dispositional mindfulness. Conversely, Indonesia’s Islamic cultural background may foster specific forms of mindful states that might differ from Western or Buddhist conceptualizations of mindfulness.

Based on the theoretical background and considerations above, this study proposes the following hypotheses:

*H1*: There will be significant differences in dispositional mindfulness levels between Chinese and Indonesian participants.

*H2*: A positive correlation exists between dispositional mindfulness and prosocial behavior in both countries.

*H3*: Empathy mediates the relationship between dispositional mindfulness and prosocial behavior.

*H4*: Cultural background (China vs. Indonesia) moderates the indirect effect of dispositional mindfulness on prosocial behavior through empathy.

By testing these hypotheses through analysis of data collected from participants in China and Indonesia, two countries with distinct cultural differences, this study’s findings may have significant implications for both theory and practice. Theoretically, it will enhance our understanding of how important psychological constructs such as mindfulness, empathy, and prosocial behavior manifest and interrelate across different cultural contexts, thereby enriching the fields of cross-cultural psychology and mindfulness research. Practically, the findings may provide guidance for designing and implementing cross-cultural mindfulness interventions, helping us better utilize mindfulness to promote prosocial behavior and social harmony across different cultural contexts.

## 2 Method

### 2.1 Participants and procedure

This study was approved by the Ethics Committee of the School of Teacher Education at Hechi University (IRB No. H2305). Using stratified random sampling based on department and grade level distribution, we recruited 683 undergraduate and graduate students from China (*N* = 357, 174 males; Mean age = 18.27 years, SD = 0.59) and Indonesia (*N* = 326, 156 males; Mean age = 24.72 years, SD = 1.16). All questionnaires were administered in English through an online platform^1^. The system required all items to be completed before submission. To ensure data quality, researchers implemented supplementary measures: providing a bilingual glossary of technical terms for reference and allowing participants to look up unfamiliar vocabulary. Cases involving prior mindfulness training or insufficient English proficiency were excluded. The final study sample comprised participants with no prior mindfulness training experience, and all participants reported high confidence in their English language abilities. Participants were instructed to carefully read the directions and complete all questionnaire items. They first signed an online informed consent form, then provided demographic information, and finally completed the questionnaires. Upon submission, participants received 5 RMB as compensation. All Indonesian participants self-reported as local Indonesians and Muslims. All Chinese participants self-reported as local Chinese with no religious affiliation (though some participants might have withheld religious information due to awareness of restrictions on religious activities in Chinese universities) and no overseas experience.

### 2.2 Measures

#### 2.2.1 Dispositional mindfulness

Dispositional mindfulness (DM) was assessed using the Mindful Attention Awareness Scale (MAAS; Brown and Ryan, 2003). The MAAS assesses individuals’ open and receptive attitude toward present experiences in daily life, including awareness of both internal sensations and external environment. The scale consists of 15 items rated on a 6-point scale (1 = almost never, 6 = almost always), with the total score indicating the level of mindfulness. A sample item is “I find it difficult to stay focused on what’s happening in the present” (reverse-scored). The Cronbach’s alpha coefficient for the total sample was 0.84 (0.80 for Chinese participants and 0.87 for Indonesian participants). Although the scale has demonstrated good internal consistency across various cultural contexts (Zainal et al., 2015; Klainin-Yobas et al., 2023; Padhi et al., 2024), measurement invariance analysis was conducted to assess the cross-cultural equivalence of MAAS in Chinese and Indonesian samples. Results indicated that MAAS achieved configural invariance (CFI = 0.77, RMSEA = 0.09, SRMR = 0.07) and metric invariance (CFI = 0.75, RMSEA = 0.09, SRMR = 0.09), but not scalar invariance. This suggests that while the basic structure of MAAS is similar across the two cultural groups, caution should be exercised when comparing group mean scores.

#### 2.2.2 Empathy

The Interpersonal Reactivity Index (IRI; Davis, 1983) is a 22-item scale comprising four dimensions: perspective taking (PT), empathic concern (EC), fantasy (FS), and personal distress (PD). Participants rated the IRI on a 5-point scale from 0 (not appropriate) to 4 (very appropriate). Total scores were calculated separately for each dimension. Sample items include “Before criticizing somebody, I try to imagine how they would feel” (PT), “After seeing a play or movie, I feel as though I were one of the characters” (FS), “I would describe myself as a pretty soft-hearted person” (EC), and “Being in a tense emotional situation scares me” (PD). Cronbach’s *α* coefficients were 0.70, 0.64, 0.67, and 0.74, respectively. For Chinese participants, Cronbach’s α coefficients ranged from 0.62 for EC to 0.77 for PD, while for Indonesian participants, they ranged from 0.43 for FS to 0.71 for PD (Table 1). Measurement invariance analysis was conducted to assess the cross-cultural equivalence of IRI in Chinese and Indonesian samples. Results indicated that IRI achieved configural invariance (CFI = 0.67, RMSEA = 0.09, SRMR = 0.10) and metric invariance (CFI = 0.66, RMSEA = 0.09, SRMR = 0.10), but not scalar invariance. This suggests that while the basic structure of IRI is similar across the two cultural groups, caution should be exercised when comparing mean scores.

**Table 1:** Correlations among research variables in China and Indonesia.

	DM	PT	FS	EC	PD	PBF	PBFr	PBS
DM	0.80/0.87							
PT	0.34^***^/0.20^***^	0.72/0.67						
FS	0.07/0.05	0.21^***^/0.38^***^	0.65/0.43					
EC	0.19^***/0^.22^***^	0.16^***^/0.50^***^	0.34^***^/0.40^***^	0.62/0.64				
PD	−0.33^***^/−0.03	−0.05/0.30^***^	0.23^***^/0.21^***^	0.06/−0.06	0.77/0.71			
PBF	0.38^***^/0.35^***^	0.36^***^/0.36^***^	0.16^***^/0.28^***^	0.39^***^/0.41^***^	−0.03/0.03	0.88/0.79		
PBFr	0.34^***^/0.33^***^	0.34^***^/0.41^***^	0.25^***^/0.32^***^	0.40^***^/0.51^***^	0.02/0.02	0.75^***^/0.78^***^	0.85/0.81	
PBS	0.32^***^/0.25^***^	0.36^***^/0.23^***^	0.23^***^/0.20^***^	0.33^***^/0.18^**^	−0.06/0.08	0.57^***^/0.68^***^	0.52^***^/0.55^***^	0.81/0.79

^**^p < 0.01, ^***^p < 0.001. Numbers after the slashes were coefficients among Indonesian participants. DM, Dispositional Mindfulness; PT, Perspective Taking; FS, Fantasy; EC, Empathic Concern; PD, Personal Distress; PBF, prosocial behavior toward family; PBFr, Prosocial Behavior toward Friends; PBS, Prosocial Behavior toward Strangers.

#### 2.2.3 Prosocial behavior

The Self-Report Altruism Scale Distinguished by the Recipient (SRAS-DR; Oda et al., 2013b) contains 21 items measuring prosocial behavior toward three types of recipients: family members (prosocial behavior toward family, PBF), friends or acquaintances (prosocial behavior toward friends, PBFr), and strangers (prosocial behavior toward strangers, PBS). Participants rated their frequency of engaging in prosocial behaviors using five categories from 1 (never) to 5 (always). Total scores for PBF, PBFr, and PBS were calculated separately and used as dependent variables. The scale has demonstrated acceptable reliability and validity among Asian participants (Oda et al., 2013a). Sample items include “I support my family members when they are not feeling well” (PBF), “I give my best wishes to friends on their birthdays” (PBFr), and “When a stranger suddenly falls ill or gets injured, I look after them or call an ambulance” (PBS). Cronbach’s *α* coefficients were 0.87 (PBF), 0.88 (PBFr), and 0.81 (PBS). For Chinese participants, α coefficients were 0.88 (PBF), 0.85 (PBFr), and 0.81 (PBS). For Indonesian participants, α coefficients were 0.79 (PBF), 0.81 (PBFr), and 0.79 (PBS). Measurement invariance analysis was conducted to assess the cross-cultural equivalence of SRAS-DR in Chinese and Indonesian samples. Results indicated that SRAS-DR achieved configural invariance (CFI = 0.83, RMSEA = 0.09, SRMR = 0.07) and metric invariance (CFI = 0.81, RMSEA = 0.09, SRMR = 0.08), but not scalar invariance. This suggests that while the basic structure of SRAS-DR is similar across the two cultural groups, caution should be exercised when interpreting group mean score differences.

#### 2.2.4 Control variables

Previous research has shown that demographic variables, particularly gender and age, significantly influence individuals’ levels of mindfulness, empathy, and prosocial behavior (Ardenghi et al., 2023; Li et al., 2024; Pastor et al., 2024). Additionally, studies have found that socioeconomic status affects both access to mindfulness practice resources and capacity for prosocial behavior (Piff et al., 2010). Based on these established relationships, this study incorporated multiple control variables, including gender, age, religious affiliation, parental education levels (father and mother), and average monthly household income, to control for potential confounding effects.

### 2.3 Statistical analysis

Data analysis was conducted using R 4.4.1 (R Core Team, 2023) and Process v4.2 (Hayes, 2022). Initially, descriptive statistics and Pearson correlation coefficients among variables were calculated, and independent samples t-tests were performed to compare differences between Chinese and Indonesian samples.

Model 4 in Process was employed to test the mediating role of empathy in the relationship between mindfulness and prosocial behavior. In this analysis, mindfulness served as the independent variable, prosocial behavior (toward family, friends, and strangers) as the dependent variable, and the four dimensions of empathy as mediating variables. Analyses were conducted separately for Chinese and Indonesian samples to explore cross-cultural differences.

Further analysis utilizing Model 59 in Process examined the moderating effect of cultural background, with country (China vs. Indonesia) as the moderating variable, investigating its moderating effects on various paths within the model. All analyses for both Model 4 and Model 59 controlled for demographic variables and employed bias-corrected bootstrapping (10,000 resamples) to estimate 95% confidence intervals for indirect effects. Indirect effects were considered significant if the confidence interval did not contain zero (Hayes, 2022). The significance level for all statistical analyses was set at *α* = 0.05.

## 3 Results

### 3.1 Comparison of scale scores between Chinese and Indonesian samples

The study compared scale scores between Chinese and Indonesian samples and reported Cohen’s d effect sizes (see Table 2). Results indicated that Chinese participants scored higher than Indonesian participants on all measured dimensions except for PD. Specifically, Chinese participants showed higher scores in DM (*d* = 0.59), PT (*d* = 0.27), FS (*d* = 0.94), EC (*d* = 0.73), and PBF (*d* = 1.13), PBFr (*d* = 1.36), and PBS (*d* = 0.66), while scoring slightly lower in PD (*d* = −0.19). However, since scalar invariance was not achieved for MAAS, IRI (including PT, FS, EC, and PD dimensions), and SRAS-DR (including PBF, PBFr, and PBS dimensions), these differences may reflect not only genuine group differences but also measurement tool variations across cultures. Notably, the fantasy (FS) dimension of IRI demonstrated low internal consistency in the Indonesian sample (*α* = 0.43), which may affect the reliability of score differences in this dimension.

**Table 2:** Mean difference between nations.

	Chinese (*N* = 357)		Indonesians (*N* = 326)		t	Cohen’s d
	*M*	SD	*M*	SD		
DM	63.41	9.57	57.36	10.82	7.75^***^	0.59
PT	18.35	3.45	17.45	3.15	3.55^***^	0.27
FS	22.56	4.08	19.21	2.98	12.30^***^	0.94
EC	22.88	3.79	20.09	3.83	9.58^***^	0.73
PD	14.72	4.27	15.45	3.39	−2.48^*^	−0.19
PBF	30.40	4.21	25.42	4.61	14.75^***^	1.13
PBFr	31.29	3.49	25.62	4.72	17.71^***^	1.36
PBS	27.30	5.29	23.95	4.85	8.63^***^	0.66

^*^p < 0.05, ^**^p < 0.01, ^***^p < 0.001. DM, Dispositional Mindfulness; PT, Perspective Taking; FS, Fantasy; EC, Empathic Concern; PD, Personal Distress; PBF, prosocial behavior toward family; PBFr, Prosocial Behavior toward Friends; PBS, Prosocial Behavior toward Strangers. Cohen’s d interpretation: |d| < 0.2 negligible, 0.2 ≤ |d| < 0.5 small effect, 0.5 ≤ |d| < 0.8 medium effect, |d| ≥ 0.8 large effect.

### 3.2 Correlations among study variables

Correlation analyses were conducted for both Chinese and Indonesian participants (Table 1). DM demonstrated significant positive correlations with two dimensions of empathy (PT and EC) and all dimensions of prosocial behavior (PBF, PBFr, and PBS) in both Chinese and Indonesian participants. All dimensions of empathy, except PD, showed positive correlations with all dimensions of prosocial behavior. An interesting finding was that DM correlated negatively with PD among Chinese participants, while this correlation was not significant among Indonesian participants.

### 3.3 Mediation analysis of dispositional mindfulness, empathy, and prosocial behavior

Results of the mediation analysis revealed differences in the relationship between DM and prosocial behavior between Chinese and Indonesian samples (see Table 3).

**Table 3:** Mediation effects in two nations.

Independent-dependent	Mediator	Nation	*β*	Boot SE	LLCI	ULCI
DM-PBF	PT	China	0.0784	0.0196	0.0423	0.1193
		Indonesia	0.0247	0.0172	−0.0002	0.0651
	FS	China	−0.0017	0.0044	−0.0121	0.0064
		Indonesia	0.0015	0.0074	−0.0138	0.0168
	EC	China	0.0564	0.0206	0.0213	0.1021
		Indonesia	0.0459	0.0046	−0.0083	0.0118
	PD	China	−0.0152	0.0163	−0.0491	0.0163
		Indonesia	0.0007	0.0046	−0.0083	0.0118
	Direct effect	China	0.1166	0.0224	0.0725	0.1607
		Indonesia	0.1110	0.0216	0.0685	0.1536
DM-PBFr	PT	China	0.0653	0.0176	0.0327	0.0189
		Indonesia	0.0278	0.0177	0.0016	0.0703
	FS	China	0.0045	0.0059	−0.0036	0.0189
		Indonesia	0.0015	0.0073	−0.0130	0.0165
	EC	China	0.0557	0.0196	0.0212	0.0976
		Indonesia	0.0658	0.0195	0.0304	0.0165
	PD	China	−0.0239	0.0167	−0.0594	0.0071
		Indonesia	0.0009	0.0044	−0.0069	0.0121
	Direct effect	China	0.0918	0.0189	0.0547	0.1289
		Indonesia	0.0993	0.0210	0.0581	0.1406
DM-PBS	PT	China	0.0750	0.0213	0.0378	0.1204
		Indonesia	0.0192	0.0243	−0.0164	0.0792
	FS	China	0.0059	0.0071	−0.0049	0.0232
		Indonesia	0.0016	0.0079	−0.0145	0.0184
	EC	China	0.0461	0.0175	0.0164	0.0840
		Indonesia	0.0066	0.0079	−0.0145	0.0184
	PD	China	0.0030	0.0177	−0.0295	0.0405
		Indonesia	−0.0009	0.0057	−0.0129	0.0125
	Direct effect	China	0.0996	0.0296	0.0413	0.1578
		Indonesia	0.0976	0.0252	0.0481	0.1471

DM, Dispositional Mindfulness; PT, Perspective Taking; FS, Fantasy; EC, Empathic Concern; PD, Personal Distress; PBF, prosocial behavior toward family; PBFr, Prosocial Behavior toward Friends; PBS, Prosocial Behavior toward Strangers.

For the Chinese sample, PT and EC demonstrated significant mediating effects between DM and all three types of prosocial behavior (PBF, PBFr, and PBS). Specifically, DM showed indirect effects through PT on PBF (*β* = 0.0784, 95% CI [0.0423, 0.1193]), PBFr (*β* = 0.0653, 95% CI [0.0327, 0.0189]), and PBS (*β* = 0.0750, 95% CI [0.0378, 0.1204]). Similarly, DM demonstrated significant indirect effects through EC on PBF (*β* = 0.0564, 95% CI [0.0213, 0.1021]), PBFr (*β* = 0.0557, 95% CI [0.0212, 0.0976]), and PBS (*β* = 0.0461, 95% CI [0.0164, 0.0840]).

Model analysis for the Chinese sample revealed that the overall model explained 32.33% of the variance in PBF (*R*^2^ = 0.3233, *F*(10, 346) = 16.5304, *p* < 0.001), 30.08% in PBFr (*R*^2^ = 0.3008, *F*(10, 346) = 14.8852, *p* < 0.001), and 25.25% in PBS (*R*^2^ = 0.2525, *F*(10, 346) = 11.6892, *p* < 0.001). Overall, the model demonstrated strongest explanatory power for PBF, followed by PBFr, and relatively weaker explanation for PBS.

In contrast, mediating effects were more limited in the Indonesian sample. PT showed significant mediation only between DM and PBFr (*β* = 0.0278, 95% CI [0.0016, 0.0703]), while EC significantly mediated only between DM and PBFr (*β* = 0.0658, 95% CI [0.0304, 0.0165]).

For the Indonesian sample, the model explained 27.94% of the variance in PBF (*R*^2^ = 0.2794, *F*(10, 315) = 12.2129, *p* < 0.001), 35.61% in PBFr (*R*^2^ = 0.3561, *F*(10, 315) = 17.4178, *p* < 0.001), and 12.04% in PBS (*R*^2^ = 0.1204, *F*(10, 315) = 4.3135, *p* < 0.001). Overall, the model showed strongest explanatory power for PBFr, followed by PBF, and relatively weaker explanation for PBS.

Notably, FS and PD did not demonstrate significant mediating effects in either sample. Furthermore, the direct effects of DM on all types of prosocial behavior remained significant in both country samples, indicating that DM influences prosocial behavior both directly and through empathy. In other words, empathy serves as a partial mediator between DM and prosocial behavior.

### 3.4 Analysis of cultural background’s moderating effect

To examine the moderating effect of cultural background (China vs. Indonesia) on the relationships among DM, empathy, and prosocial behavior, moderated mediation analysis was conducted (Table 4). Results revealed that cultural background primarily moderated the indirect effect of DM on PBS through PT (index = −0.0360, 95% CI [−0.0666, −0.0055]). This finding suggests that the effect of DM in promoting PBS through enhanced perspective-taking ability was stronger in the Chinese sample compared to the Indonesian sample.

**Table 4:** Moderated mediation effects in two nations.

Independent-Dependent	Mediator	Index	Boot SE	LLCI	ULCI
DM-PBF	PT	−0.0177	0.0137	−0.0431	0.0108
	EC	−0.0027	0.0132	−0.0239	0.0277
DM-PBFr	PT	−0.0038	0.0141	−0.0289	0.0268
	EC	0.0176	0.0136	−0.0090	0.0448
DM-PBS	PT	−0.0360	0.0157	−0.0666	−0.0055
	EC	−0.0191	0.0132	−0.0455	0.0070

DM, Dispositional Mindfulness; PT, Perspective Taking; EC, Empathic Concern; PBF, prosocial behavior toward family; PBFr, Prosocial Behavior toward Friends; PBS, Prosocial Behavior toward Strangers.

However, cultural background did not significantly moderate the indirect effects of DM on PBF and PBFr through either PT or EC. Similarly, cultural background did not significantly moderate the indirect effect of DM on PBS through EC. The 95% confidence intervals for these results contained zero, indicating that these moderating effects were not statistically significant.

**Figure 1:**
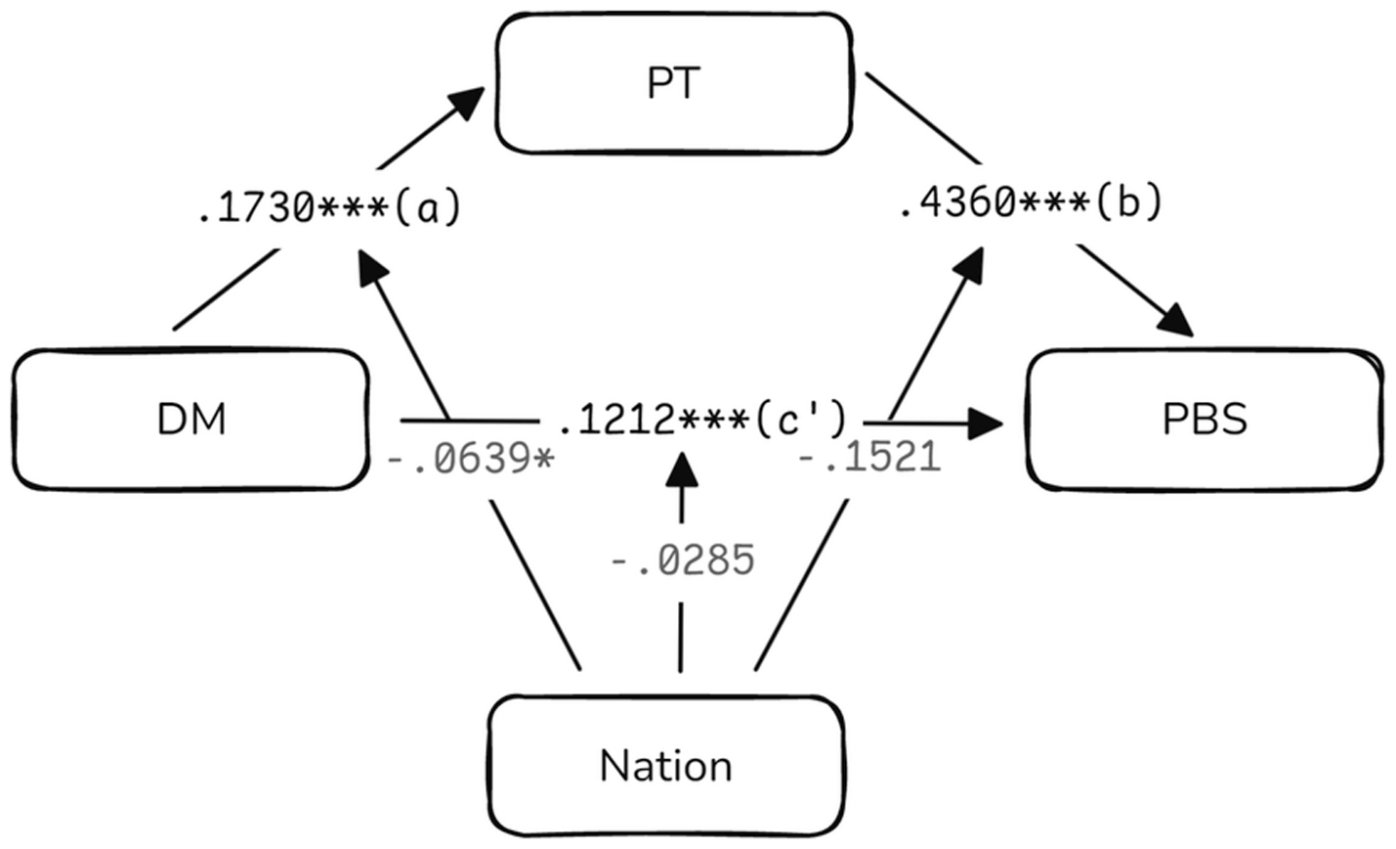
Moderated mediation model of dispositional mindfulness, perspective taking, and prosocial behavior toward strangers. DM, Dispositional Mindfulness; PT, Perspective Taking; PBS, Prosocial Behavior toward Strangers; Nation, Country (moderator variable). Standardized path coefficients are shown. Path a represents the effect of DM on PT, path b represents the effect of PT on PBS, and path c’ represents the direct effect of DM on PBS. *p < 0.05, ***p < 0.001.

To more clearly illustrate the relationships among DM, PT, and PBS, as well as the moderating role of cultural background in these relationships, a model diagram was created (see Figure 1). The diagram depicts the structural relationships with DM as the independent variable, PBS as the dependent variable, PT as the mediating variable, and country as the moderating variable. The path coefficients in the model diagram reflect the direct and indirect effects between variables.

**Figure 2:**
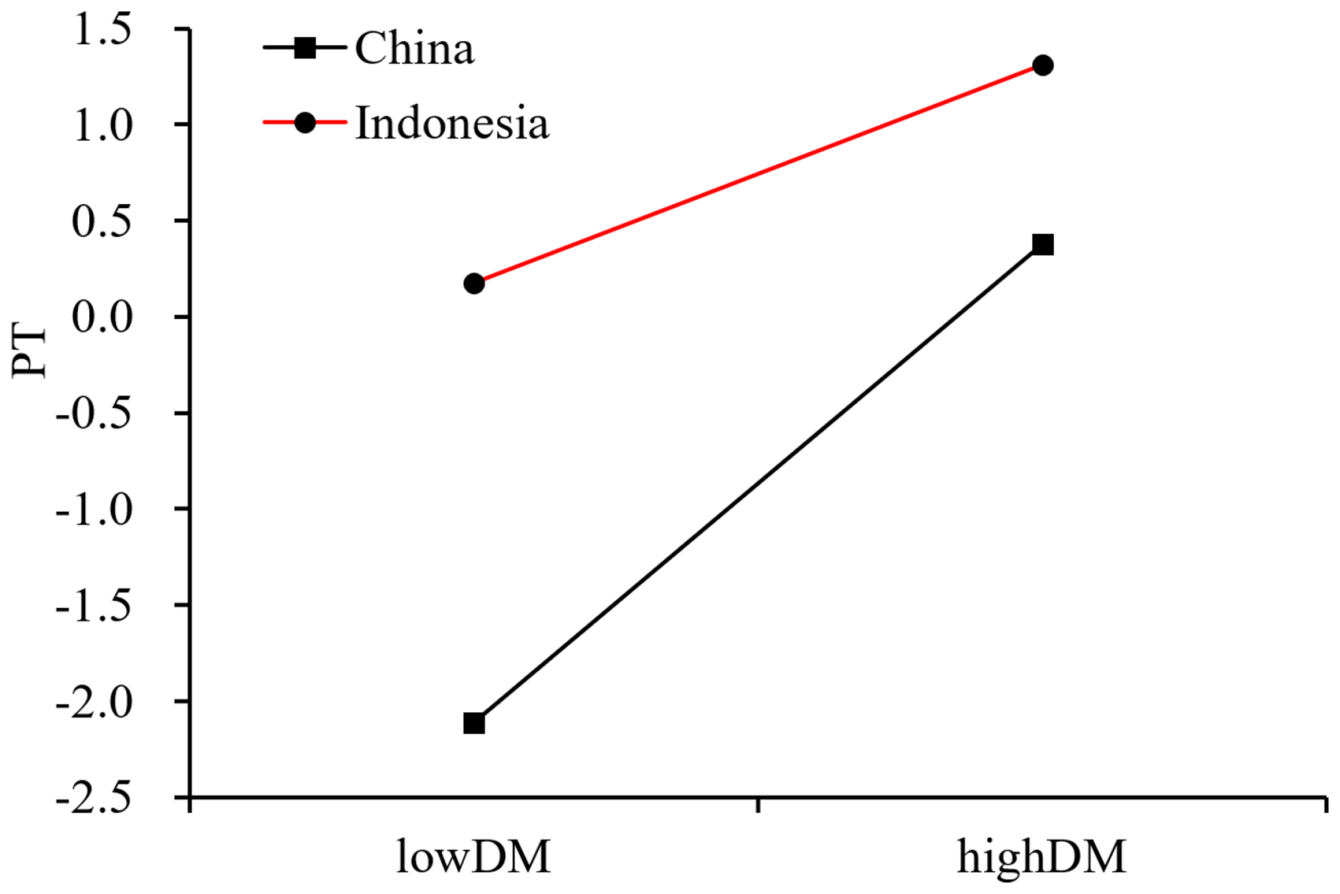
The moderating effect of cultural background on the relationship between dispositional mindfulness and perspective Taking. DM, Dispositional Mindfulness; PT, Perspective Taking.

To further examine the moderating effect of cultural background on the relationship between DM and PT, simple slope analysis was conducted (see Figure 2). Results revealed that the positive relationship between DM and PT was significant in both Chinese and Indonesian samples, albeit with different magnitudes. Specifically, in the Chinese sample, the relationship between DM and PT was stronger (*β*_simple_ = 0.1173, *p* < 0.001). In contrast, while still significant, this relationship was weaker in the Indonesian sample (*β*_simple_ = 0.0534, *p* < 0.01). This interaction effect was significant (Δ*R*^2^ = 0.0091, *F*(1, 674) = 6.8696, *p* < 0.01), indicating that cultural background indeed moderated the relationship between DM and PT.

## 4 Discussion

### 4.1 Cultural differences in dispositional mindfulness levels

The study found that Chinese participants scored higher on the MAAS scale than Indonesian participants (*d* = 0.59), superficially supporting the first research hypothesis. However, interpreting these differences requires moving beyond singular cultural or religious attributions. While some researchers suggest that Buddhism has been implicitly integrated into Chinese people’s thoughts, emotions, and behaviors, with years of Buddhist cultural influence potentially fostering higher DM levels in the region (Chau, 2010; Qin and Song, 2020), our findings indicate the need for a more nuanced and critical perspective.

First, potential measurement bias must be considered. Although the MAAS scale demonstrated good internal consistency in both samples, it failed to achieve scalar invariance, suggesting that direct comparisons of mean scores between the groups may be problematic. This finding echoes the research of Feng et al. (2018), who noted that many psychological measurement tools might face equivalence issues in cross-cultural applications. Therefore, score differences cannot be simply equated with actual differences in DM levels between the two countries. Second, even if actual differences exist, the causes are likely multifaceted. For instance, differences between China and Indonesia in religious beliefs, educational systems, social structures, and life rhythms are all sociocultural factors that may shape individual mindfulness experiences and expressions, subsequently influencing DM levels (Grossman and Dam, 2013).

### 4.2 The mediating mechanism of empathy between dispositional mindfulness and prosocial behavior

Our findings support H2, confirming significant positive correlations between DM and prosocial behavior in both country samples. Moreover, empathy was found to mediate the relationship between DM and prosocial behavior (H3), though this mediating effect exhibited interesting cultural variation patterns. Specifically, in the Chinese sample, both PT and EC significantly mediated the relationship between DM and various types of prosocial behavior (PBF, PBFr, and PBS); while in the Indonesian sample, the two dimensions of empathy (PT and EC) showed significant mediating effects only in the context of friend relationships. Berry et al. (2018) found that DM can promote prosocial behavior by enhancing affective empathy (empathic concern), while our study further refined this mechanism by revealing that cognitive empathy (perspective taking) plays an equally important role. This finding aligns with Trautwein et al.'s (2014) view that mindfulness can enhance both cognitive and affective empathy simultaneously.

Several explanations can be proposed for these findings: First, as Davis (1983) noted, the fantasy dimension primarily reflects an individual’s capacity to engage with fictional situations, which, although related to empathy, may be somewhat removed from actual prosocial behavior. Berninger (2018) research suggests that empathetic experiences in fictional contexts may involve different psychological mechanisms than prosocial responses in real life. Second, studies have found that mindfulness training primarily enhances awareness of present real situations rather than imaginative engagement (Farb, 2012; Kohls et al., 2019). This suggests that DM may more directly influence dimensions closely related to present experience, such as PT and EC, while having relatively weaker effects on imaginative engagement. Additionally, from a cultural perspective, East Asian cultures may emphasize actual interpersonal interactions over emotional investment in fictional situations (Ishii and Eisen, 2016; Yang, 2024), which partly explains why no mediating effect of FS was observed in the Chinese sample.

Notably, PD did not demonstrate significant mediating effects in this study. This may be related to the core mechanisms of mindfulness. Research indicates that mindfulness training can enhance emotional regulation abilities and reduce excessive arousal and anxiety responses when facing others’ difficulties (Birnie et al., 2010; Raugh and Strauss, 2024). In other words, mindfulness practice can help individuals maintain awareness while avoiding emotional over-involvement (Cho, 2024). In fact, this mechanism may have actually reduced the mediating role of personal distress in the relationship between DM and prosocial behavior. As Klimecki et al. (2014) emphasized, effective prosocial behavior requires balancing empathy and emotional regulation: excessive personal distress may actually hinder effective helping behavior. Our findings suggest that DM may more effectively promote prosocial behavior by reducing PD levels while enhancing PT and EC.

These findings collectively point to a more nuanced theoretical framework: DM selectively influences different types of empathic responses—enhancing adaptive empathy dimensions (PT and EC) while regulating or reducing dimensions that might impede effective prosocial behavior (such as PD)—ultimately promoting prosocial behavior. This discovery not only deepens our understanding of the DM-empathy-prosocial behavior relationship but also provides clearer directions for future research and practice.

### 4.3 The moderating role of cultural background

This study found that cultural background moderated the relationships among DM, empathy, and prosocial behavior only under specific conditions, partially supporting our fourth hypothesis. Specifically, cultural background significantly moderated the indirect effect of DM on PBS through PT, with this effect being stronger in the Chinese sample. From a cognitive processing perspective, perspective taking, as a form of cognitive empathy, requires individuals to actively suppress their own viewpoint and adopt others’ perspectives (Wu and Keysar, 2007). Varnum et al. (2010) suggest that individuals from East Asian cultural backgrounds, which emphasize interdependence, may perform better in perspective-taking tasks. This advantage may be particularly evident when interacting with strangers, as understanding strangers’ perspectives requires more cognitive effort compared to understanding family members and friends.

Notably, cultural background did not demonstrate significant moderating effects in most cases. This might suggest that the core mechanisms of mindfulness possess cross-cultural universality. Therefore, significant moderating effects may only emerge when specific cognitive processing (such as perspective taking) is highly correlated with cultural characteristics. Of course, this might also reflect limitations in the study’s design regarding the detection of subtle cultural differences, warranting further validation by future researchers.

### 4.4 Theoretical contributions and practical implications

This study provides a more nuanced and in-depth understanding of the relationships among DM, empathy, and prosocial behavior, while also offering new perspectives on mindfulness research across cultural contexts. Our findings challenge the existing view that DM has uniform effects on all types of empathy (Greeson et al., 2015), while supporting other perspectives, such as the potential cross-cultural stability of DM in promoting prosocial behavior (Luengo Kanacri et al., 2020).

At the practical level, our findings provide important implications for mindfulness interventions in cross-cultural settings. First, in mental health practice, mindfulness training can be utilized as an intervention method for enhancing empathy and promoting prosocial behavior. Specifically, based on our findings, targeted mindfulness exercises can be designed to enhance PT and EC. Second, when designing mindfulness programs for individuals from different cultural backgrounds, special attention may need to be paid to cultivating perspective-taking abilities, particularly in promoting PBS. Finally, mindfulness practices should be adapted to participants’ cultural worldviews, with more targeted mindfulness intervention programs designed to promote the improvement of various social relationships across different cultural contexts.

### 4.5 Limitations and future directions

While this study provides valuable insights, several limitations should be acknowledged. First, the cross-sectional design limits our ability to make causal inferences. Second, the sample being restricted to university students may affect the generalizability of the results. Additionally, issues with the cross-cultural equivalence of measurement tools may have impacted the precision of result interpretation.

Based on these limitations and our findings, several directions for future research warrant consideration. First, there is a need to develop and validate more culturally sensitive DM and empathy measurement tools for non-Western cultural contexts. Longitudinal or experimental research designs are needed to better explore the causal relationships among DM, empathy, and prosocial behavior. Furthermore, expanding the sample to include participants from different age groups, occupations, and socioeconomic backgrounds would enhance the universality of research findings. Deeper investigation into how cultural factors (such as individualism–collectivism orientation) moderate the effects of DM could be valuable. Additionally, cross-cultural intervention studies comparing the effectiveness of mindfulness practices across different cultural contexts would provide valuable insights. As Kirmayer (2015) emphasized, interdisciplinary collaboration is crucial for comprehensively understanding the cultural dimensions of mindfulness. These suggested directions would not only address the current study’s limitations but also advance our understanding of how mindfulness operates across different cultural contexts.

## Data Availability

The raw data supporting the conclusions of this article will be made available by the authors, without undue reservation.

## Footnotes

^1^ www.sojump.com
